# Strengthening effective preventive services for refugee populations: toward communities of solution

**DOI:** 10.1186/s40985-018-0082-y

**Published:** 2018-02-07

**Authors:** Kim S. Griswold, Kevin Pottie, Isok Kim, Wooksoo Kim, Li Lin

**Affiliations:** 1 Department of Family Medicine, Jacobs School of Medicine and Biomedical Sciences, University at Buffalo, The State University of New York, 77 Goodell St., Buffalo, NY 14203 USA; 2 Departments of Family Medicine and Epidemiology and Community Medicine, University of Ottawa, Stewart Street, Ottawa, ON K1N 6N5 Canada; 3 Bruyère Research Institute, 85 Primrose Ave, Annex E-208, Ottawa, ON K1R 6M1 Canada; 4 School of Social Work, University at Buffalo, The State University of New York, Buffalo, USA; 5 Department of Industrial and Systems Engineering, University at Buffalo, The State University of New York, Buffalo, USA; 6 University of Ottawa, 75 Bruyère St, Ottawa, ON K1S 0P6 Canada

**Keywords:** Refugees, Preventive care, Screening, Health care disparities, Health insurance, Public health, Primary care

## Abstract

Refugee populations have unequal access to primary care and may not receive appropriate health screening or preventive service recommendations. They encounter numerous health care disadvantages as a consequence of low-income status, race and ethnicity, lower educational achievement, varying degrees of health literacy, and limited English proficiency. Refugees may not initially embrace the concept of preventive care, as these services may have been unavailable in their countries of origin, or may not be congruent with their beliefs on health care. Effective interventions in primary care include the appropriate use of culturally and linguistically trained interpreters for health care visits and use of evidence-based guidelines. Effective approaches for the delivery of preventive health and wellness services require community engagement and collaborations between public health and primary care. In order to provide optimal preventive and longitudinal screening services for refugees, policies and practice should be guided by unimpeded access to robust primary care systems. These systems should implement evidence-based guidelines, comprehensive health coverage, and evaluation of process and preventive care outcomes.

## Background

Refugee populations have unequal access to primary care and may not receive appropriate health screening or preventive service recommendations [1, 2]. Refugees encounter numerous structural and process barriers as a consequence of low-income status, race and ethnicity, varying degrees of health literacy, and limited English proficiency (LEP) [3]. Global migration strategy lacks consistent and equitable policies addressing public health insurance and health assessment for refugees [4]. These risk factors contribute to health care disparities for resettled refugee populations [5].

## Medical screening for resettled refugees

Epidemiology and public health principles inform policy and can help develop responsive health systems [6]. Newly resettled refugees, for example, undergo a medical screening process which typically focuses on communicable diseases such as tuberculosis, HIV, and hepatitis [7].

A recent review of screening procedures in selected high-resource countries (North America, Europe, and Australia/New Zealand) revealed a strong focus on infectious disease screening. Few guidelines described cancer screening or screening for chronic diseases, such as hypertension or diabetes, although studies have shown high rates of chronic conditions among resettled refugees [8, 9].

## Health assessments

Pre-existing risk factors and chronic conditions may not be appropriately addressed in medical screening due to issues of cost, lack of continuity of care, and/or lack of adequate insurance coverage [10, 11]. While medical screening often has an infectious disease focus, in recent years, health assessments have begun to emerge with a community primary health care perspective [3] (see Table 1). Health assessments also often utilize community engagement strategies when addressing preventive care.

**Table 1:** Integrating public health and primary care

Tailor the promotion and delivery of various preventive and screening measures to specific refugee populations.	
Integrate data systems to improve estimates of health services and health outcomes.	
Utilize social determinants of health for local and regional guidelines.	
Enhance training of epidemiologists in policy delivery and immigrant community engagement and enhance public health and primary care collaborations.	
Based on Bocanegra, et al. [6]	

For example, a Canadian evidence-based guideline recommended screening high risk refugees for chronic conditions including cervical cancer, diabetes, and depression but also for dental disease and visual acuity. These health assessment interventions were intended for primary care practitioners who would be developing a therapeutic relationship with refugees within community practices [3]. Nevertheless, there were still challenges in implementing effective counseling for diverse diets and new medication across cultural and linguistic barriers.

Health assessments of newly resettled refugees often occur in primary care settings and incorporate preventive care and provision of cancer screening (Table 2).

**Table 2:** Preventive care resources for practitioners caring for refugees

*U.S. Centers for Disease Control *https://www.cdc.gov/cancer/dcpc/prevention/screening.htm*	
*Office of Refugee Resettlement, U.S. Department of Health and Human Services *https://www.acf.hhs.gov/orr*	
*Canadian Collaboration for Immigrant and Refugee Health *www.ccirhken.ca*	
*Australasian Society for Infectious Diseases and Refugee Health Network of Australia *http://ccirhken.ca/ccirh_main/wp-content/uploads/2016/03/ASID-RHeANA-Refugee-Guidelines-2016.pdf*	

In a Delphi consensus paper, use of evidence-based guidelines emerged as a priority implementation resource to support “personalized” and comprehensive care of refugees [12]. Refugees, however, carry lived experience and structural barriers to health care and thus often show lower rates of preventive and cancer screening [1, 9]. For example, in one study assessing the use of these services, patients from Somalia had lower rates of recommended vaccines and gynecological and gastrointestinal screening interventions in contrast to non-Somali participants [13].

## Structural and process barriers to preventive care and cancer screening

Health insurance is critical to address significant structural barriers to primary care, medication, and other services. A comprehensive study looking at refugees resettled in Germany found that care access restrictions “may have ultimately increased costs e.g. due to delayed care, focus on treatment of acute conditions instead of prevention and health promotion…” [14]. In a US report on racial and ethnic disparities in care, primary care is emphasized as requisite to the provision of preventive health services; under-insurance or lack of insurance reduces access to care and uninsured patients have “much lower” rates of preventive care [3, 15]. Medicaid expansion under the Affordable Care Act in Oregon significantly improved the provision of preventive services [16].

Limited English  proficiency (LEP) has a deleterious effect on care access as well as the receipt or uptake of preventive services. A large longitudinal study in Canada showed that LEP correlates with reduced health status beginning shortly after arrival in the host country [17]. In another study among US Hispanics, Spanish-speaking Hispanics had less access to health care and lower rates of receiving preventive services when compared to English-speaking Hispanics [18]. Similarly, in a study of Asian-Americans (Chinese and Vietnamese), language-discordant interviews resulted in lower rates of health education and improved with the use of an interpreter [19].

Health literacy—the ability to obtain, process, and understand basic health information—is a determinant of an individual’s overall health and health care utilization. Low health literacy is an important barrier to quality preventive care. Lack of knowledge of the healthcare system is a significant contributor to health disparities for low income native-born citizens but has more adverse effects on healthcare for refugees with limited language proficiency [20]. Many refugees struggle initially to understand health insurance and the concept of chronic disease [21].

Cultural, religious, and traditional beliefs may slow refugees’ uptake of our “scientific” concept of preventive care, as these services may not be available in their countries of origin or culturally congruent with their perspectives on health care [22]. Language and communication barriers, refugees’ values toward preventive care, and poor patient-physician relationships impede preventive screening and counseling [23]. Refugees, fleeing countries in conflict, may not have had access to preventive services or information on preventive health care due to disruptions in medical infrastructure [24].

## Achieving equity for refugees in communities working toward solutions

Innovative and necessary health care interventions through primary care can mitigate some of the disparity that refugees face in receiving cancer screening and preventive and wellness care. Key strategies to improve the delivery of care for refugees include the appropriate use of culturally and linguistically trained interpreters for health care visits, consistent use of evidence-based prevention and cancer screening guidelines, and enhanced community engagement [12]. Vibrant communities working toward solutions include an “integration of strategies to promote community wellness**…** utilization of community-wide data and information systems, generation of safe and achievable living and employment situations, and advocacy with and for vulnerable populations” [25, 26].

Models of primary care that emphasize “health” and related social determinants of health versus medical contexts may be better designed to facilitate prevention guidelines [27].

Community-based education and evaluation of refugee breast health knowledge may lead to better mammography rates [28]. In addition, community-oriented methods for prevention and early intervention may enhance knowledge of mental health and its care options, while supporting the process of resettlement in a new country [29]. Another dimension to facilitate the use of preventive services by refugees may be the collaboration with non-health centric disciplines. For example, working with refugees within communities, inter-professional teamwork might employ engineering expertise to map routes to health care through phone applications, such as “WIRED-UK” [30]. A review on diabetes screening showed the importance of language congruent community health workers for developing diabetes outreach programs in underserviced neighborhoods [31]. Communication, cultural beliefs, and expectations play important roles at all levels of health and prevention.

## Conclusions

To overcome structural and process barriers and to provide effective preventive services for refugees, policies and practice should ensure access to robust innovative primary care systems, ensure comprehensive health coverage for all refugees, implement evidence-based health assessment guidelines, and study process and outcome evaluations. These efforts should come from within and beyond the walls of primary care clinics, for example engaging the leaders in refugee communities. Finally, collaborative, interdisciplinary training approaches can better prepare public health and primary health care professionals.
